# Differential gene expression in iPSC-derived human intestinal epithelial cell layers following exposure to two concentrations of butyrate, propionate and acetate

**DOI:** 10.1038/s41598-022-17296-8

**Published:** 2022-08-17

**Authors:** Menno Grouls, Aafke W. F. Janssen, Loes P. M. Duivenvoorde, Guido J. E. J. Hooiveld, Hans Bouwmeester, Meike van der Zande

**Affiliations:** 1 Division of Toxicology, Wageningen University, Wageningen University and Research, Postbus 8000, 6700 EA Wageningen, The Netherlands; 2 Wageningen Food Safety Research, Wageningen University and Research, Wageningen, The Netherlands; 3 Division of Human Nutrition, Wageningen University, Wageningen University and Research, Wageningen, The Netherlands

**Keywords:** Pluripotent stem cells, Bacterial host response, Cell signalling, Genomics, Gastrointestinal models

## Abstract

Intestinal epithelial cells and the intestinal microbiota are in a mutualistic relationship that is dependent on communication. This communication is multifaceted, but one aspect is communication through compounds produced by the microbiota such as the short-chain fatty acids (SCFAs) butyrate, propionate and acetate. Studying the effects of SCFAs and especially butyrate in intestinal epithelial cell lines like Caco-2 cells has been proven problematic. In contrast to the in vivo intestinal epithelium, Caco-2 cells do not use butyrate as an energy source, leading to a build-up of butyrate. Therefore, we used human induced pluripotent stem cell derived intestinal epithelial cells, grown as a cell layer, to study the effects of butyrate, propionate and acetate on whole genome gene expression in the cells. For this, cells were exposed to concentrations of 1 and 10 mM of the individual short-chain fatty acids for 24 h. Unique gene expression profiles were observed for each of the SCFAs in a concentration-dependent manner. Evaluation on both an individual gene level and pathway level showed that butyrate induced the biggest effects followed by propionate and then acetate. Several known effects of SCFAs on intestinal cells were confirmed, such as effects on metabolism and immune responses. The changes in metabolic pathways in the intestinal epithelial cell layers in this study demonstrate that there is a switch in energy homeostasis, this is likely associated with the use of SCFAs as an energy source by the induced pluripotent stem cell derived intestinal epithelial cells similar to in vivo intestinal tissues where butyrate is an important energy source.

## Introduction

The interactions between the host intestinal mucosa and the intestinal microbiota is a mutualistic relationship that is important for maintaining the host’s health. The human intestine offers a niche for many bacteria to live in, and in turn these bacteria fulfil an important role in metabolism of drugs and food related chemicals, and synthesis of beneficial chemicals like vitamins^1^. Upon microbial fermentation of dietary fibers, the intestinal microbiota produces short chain fatty acids (SCFAs) which are fatty acids with fewer than six carbon atoms^2,3^. The total concentration of SCFAs in the intestinal tract ranges from ~ 11 to 13 mM in the distal small intestine to 50–100 mM in the colon^3^.

Butyrate, acetate and propionate are three of the most abundantly produced intestinal SCFAs. These SCFAs serve as a prominent energy source in various cell types, and have been shown to regulate immune responses in the gut by affecting immune related signalling pathways in epithelial cells^4,5^, which in turn affect immune cells residing in the lamina propria such as monocytes (i.e. reduction in migration) and macrophages (i.e. reduction of IL-12 excretion and induction of IL-10 excretion)^6,7^. These SCFAs have also been shown to affect the barrier function of the intestinal epithelium by modulating tight junction formation^8^ and by adjusting proliferation and differentiation of intestinal epithelial cells^9,10^. Butyrate is the most studied SCFA, it maintains gastrointestinal health by serving as the main energy source for enterocytes, enhancing epithelial barrier integrity and by inhibiting inflammation^11^.

A disbalance in intestinal SCFA levels has been associated with a dysbiosis in the intestinal microbiota and has been linked to a still increasing number of diseases such as Inflammatory Bowel Disease (IBD)^12^, Alzheimer’s disease^13^ and Parkinson’s disease^14,15^. In IBD patients, the concentrations of SCFAs in the intestine are decreased due to a decrease in the number of butyrate producing bacteria that make up the microbiota^12,16,17^. The relation of SCFAs with Alzheimer’s disease is multifactorial encompassing five aspects; epigenetic regulation, modulation of neuroinflammation, maintenance of the blood–brain barrier, regulation of brain metabolism and interference in amyloid protein formation with butyrate being the most impactful SCFA^13^. For Parkinson’s disease it has been observed that changes in the makeup of the gut microbiota, in particular a decrease in SCFA producing fiber degrading bacterial strains, correlate with disease progression^14^.

Several human intestinal cell models have been used to study the mode of action of SCFAs. Initially, well-characterized conventional cell-line based models like Caco-2 cells and their coculture variants (i.e. Caco-2 and HT29 cells) were exposed to SCFAs to study their effects^18,19^. However, these are cancer cells and the effects of SCFAs on these cells cannot be easily extrapolated to human in vivo conditions as cancer cells do not use butyrate as their energy source. This leads to a build-up of butyrate and is referred to as the butyrate paradox^20–23^. Novel directions in research have been made possible after the hallmark success of culturing primary intestinal tissues and adult stem cell-based 3D human intestinal organoids (HIOs) in vitro. Subsequently, induced pluripotent stem cell (iPSC) based 3D HIOs were developed^24^. iPSCs can be differentiated into a large variety of cells in the human body by applying growth factors during their development. Using a protocol for differentiation into HIOs resulted in a microarchitecture and cell type composition similar to the human intestine. iPSC derived HIOs consist of enterocytes, goblet cells, mesenchymal and enteroendocrine cells^25–28^. Furthermore, iPSC derived HIOs have shown improved cytochrome P450 and transporter expression in comparison with Caco-2 cells^29,30^. More recently, iPSCs have been used to create 2D intestinal epithelial cell (IEC) layers that emulate the full cellular complexity of the 3D HIOs, but have an easily accessible apical and basolateral side in comparison with their 3D HIO variants which are closed spheres. This difference makes it easier to perform relevant experiments as compounds can be added to the apical side which represents the lumen, there is no build-up of cellular debris near the cells, cells are not encompassed in extracellular matrix providing free access and samples opportunities.

3D HIOs and 2D IEC layers have yet been used very limitedly to study the effect of SCFAs and predict potential responses in vivo. An early study in 2014 reported effects of SCFAs and products generated by two abundant microbiota species *Akkermansia muciniphila* and *Faecalibacterium prausnitzii* on adult stem cell derived mouse intestinal organoids. The authors showed that exposure induced cellular lipid metabolism, cell growth and cell survival pathways^31^. More recently, a limited gene expression study using qPCR analysis was performed on mouse and human adult stem cell based intestinal organoids and IEC layers, which were exposed to butyrate, propionate or acetate (at 1 and 10 mM for 24 h). It was shown that propionate and butyrate affected cell differentiation and expression of selected genes^32^.

In this study, we aimed to assess the effects of the SCFAs butyrate, propionate and acetate on human iPSC derived 2D IEC layers on a whole genome gene expression level. To this end, IEC layers were exposed to butyrate, propionate, and acetate at 1 and 10 mM for 24 h. To study the effects on a whole genome gene expression level, RNA was extracted after exposure and sequenced using next-generation sequencing. Data analysis was done at an individual gene level and at a pathway level using Gene Set Enrichment Analysis (GSEA). The present study provides novel insights into the effects of butyrate, acetate and propionate on whole genome gene expression profiles and their differences, and on the applicability of human iPSC derived IEC layers for SCFA research.

## Materials and methods

### Human induced pluripotent stem cell culture

The human iPSC cell line (CS83iCTR-33n1) was provided by the Cedars-Sinai Medical Center’s David and Janet Polak Foundation Stem Cell Core Laboratory. These cells were established through episomal reprogramming of fibroblasts of a 31-year-old healthy female. The cell line has been fully characterized and no karyotype abnormalities have been found. Undifferentiated human iPSCs were cultured in feeder free conditions using mTeSR1 medium (Stem Cell Technologies, Vancouver, Canada) on human embryonic stem cell qualified matrigel (Corning, New York, NY). For passage, iPSC colonies were dissociated using gentle cell dissociation reagent (Stem Cell Technologies).

### Human induced pluripotent stem cell differentiation into intestinal epithelial cell layers

For differentiation, human iPSCs were dissociated into single cells using accutase (Stem Cell Technologies) and cultured on human embryonic stem cell qualified matrigel-coated 24-well plates in mTeSR1 supplemented with 10 µM Y-27632 (Stem Cell Technologies) for 1 day. Human iPSCs were subsequently differentiated into definitive endoderm (DE) by incubation in RPMI1640 medium containing 1% non-essential amino acids (Gibco) and 100 ng/ml Activin A (Cell Guidance Systems, Cambridge, UK), with increasing concentrations of fetal bovine serum (0%, 0.2% and 2% on day 1, 2 and 3, respectively). 15 ng/ml BMP4 (R&D Systems, Minneapolis, MN) was also added during the first day of definitive endoderm formation. Differentiation into intestinal stem cells was subsequently induced by changing medium into DMEM/F12 containing 2% fetal bovine serum, 1% GlutaMAX (Gibco) and 250 ng/ml FGF2 (R&D Systems). After 4 days, cells were dissociated using Accutase and seeded onto polyester transwell inserts (0.4 µm, Corning) coated with Growth Factor Reduced matrigel. Cells were cultured for 7 days in intestinal differentiation medium (DMEM/F-12 containing 2% fetal bovine serum, 1% non-essential amino acids (Gibco), 1 × B27 (Gibco), 1 × N2 (Gibco), 1% penicillin/streptomycin (Gibco) and 2 mM L-glutamine (Gibco)) supplemented with 20 ng/ml EGF (R&D Systems) and 1 mM 8-Br-cAMP (Sigma). The ROCK inhibitor, y-27632 (final concentration 10 µM; Stem Cell Technologies), was added during the initial 24 h after seeding to reduce cell death. The cells were subsequently cultured for 12 days in intestinal differentiation medium supplemented with 20 ng/ml EGF, 500 µM IBMX (Sigma), 5 µM 5-aza-2′-deoxycytidine (Sigma), 20 µM PD98059 (Stem Cell Technologies), and 0.5 µM A-83-01 (Sigma). Medium was refreshed every 2–3 days.

### RNA isolation and sequencing

After the last day of differentiation cells were exposed to sodium-butyrate, sodium-propionate and sodium-acetate (Sigma-Aldrich, Steinheim, Germany) at 1 and 10 mM in triplicate (*n* = 3). After the 24 h incubation the cells were washed with 100µL DMEM^+^. After that, 350µL RA1 lysis buffer was added and incubated for 5 min. The entire volume of RA1 solution was collected as cell lysate and total RNA. Total RNA from the iPSC derived IECs was extracted using the NucleoSpin RNA isolation kit (Macherey–Nagel, Duren, Germany) according to manufacturer’s instructions. Quality of the RNA was checked using a NanoDrop One (ThermoFisher, Wilmington, Delaware, USA) and bioanalyzer (Agilent). Samples (n = 3 per treatment) were sent to BGI (Hong Kong) for transcriptome sequencing using the DNBSEQ Technology Platform with 20 M reads per sample. All the generated raw sequencing reads were filtered, by removing reads with adaptors, reads with more than 10% of unknown bases, and low quality reads. Clean reads were then obtained and stored as FASTQ format.

### Raw data processing

The RNA-seq reads were used to quantify transcript abundances. To this end the tool *Salmon*^33^ (version 1.3.0) was used to map the sequencing reads to the GRCh38.p13 human genome assembly-based transcriptome sequences as annotated by the GENCODE consortium (release 35)^34^. The obtained transcript abundance estimates and lengths were then imported in R using the package *tximport* (version 1.18.0)^35^, scaled by average transcript length and library size, and summarized on the gene-level. Such scaling corrects for bias due to correlation across samples and transcript length and has been reported to improve the accuracy of differential gene expression analysis^35^. Differential gene expression was determined using the package *limma* (version 3.46.0)^36^ using the obtained scaled gene-level counts. Briefly, before statistical analyses, counts for only protein-coding genes were retained, whereafter nonspecific filtering of the count table was performed to increase detection power^37^, based on the requirement that a gene should have an expression level greater than 10 counts, that is, 0.5 count per million reads (cpm) mapped, for at least three libraries across all 21 samples. These three libraries did not have to be within a single treatment group. Differences in library size were adjusted by the trimmed mean of M-values normalization method^38^. Counts were then transformed to log-cpm values and associated precision weights, and entered into the *limma* analysis pipeline^39^. GEO accession number: “GSE200309”.

### Data analysis at the individual gene level

Differentially expressed genes were identified by using generalized linear models that incorporate empirical Bayes methods to shrink the standard errors towards a common value, thereby improving testing power^36,40^. Genes were defined as significantly changed when *FDR* < 0.05. Venn diagrams, showing the differentially expressed genes were made using *Venny* 2.1^41^. The top 500 genes with the most variation were selected by using the interquartile range (IQR) as a measure, which is the difference between the 25th and 75th percentile. The coefficient of variation, which measures how much the values differ within a treatment, was set to 0.5 to select for differential gene expression that was related to the treatment instead of at random. Data was standardized by converting values to a z-score which sets the average to 0 and scales the other values from – 2 to 2. A principle component analysis (PCA) was performed using the library *PCAtools* on the top 500 differentially expressed genes (see selection process of the genes below)^42^. Subsequently, all individual samples were hierarchically clustered based on of the top 500 differentially expressed genes and presented in a heatmap using *heatmapper*^43^.

### Characterization of cellular makeup of IEC layers

To characterize the cellular makeup of the IEC layers and to evaluate the effects of the SCFAs on differentiation of the IECs, gene expression data of cell type specific markers and differentiation markers was analysed. For characterization of the cellular makeup, markers were obtained from studies using single cell surveys looking for cell type specific markers. In the two enterocyte sets the proximal set represents the duodenum and jejunum while the distal set represents the ileum^44^. The gene expression data of the individual control samples was pooled and gene expression was given in log2(cpm) values. For evaluation of the effects of the SCFAs on differentiation of the IECs, markers were selected from literature performing single-cell surveys on cells extracted from the intestine^44,45^ and Kyoto Encyclopedia of Genes and Genomes (KEGG) cell type signature sets. Log2(cpm) values of individual samples were averaged per exposure group and compared with the control group.

### Data analysis at the biological pathway level

Changes in gene expression were related to biologically meaningful changes using gene set enrichment analysis (GSEA). It is well accepted that GSEA has multiple advantages over analyses performed on the level of individual genes^46–48^. GSEA evaluates gene expression on the level of gene sets that are based on prior biological knowledge, GSEA is unbiased, because no gene selection step (fold change and/or p‐value cut-off) is used. A GSEA score is computed based on expression of all genes in the gene set and allows for the detection of affected biological processes that are due to only subtle changes in expression of individual genes. Gene sets were retrieved from the expert‐curated KEGG database^48^ (BRITE Functional Hierarchy level 1). Six level-1 KEGG categories were included in the analysis, metabolism, genetic information processing, environmental information processing, cellular processes, organismal system and human diseases Moreover, only gene sets comprising more than 15 and fewer than 500 genes were taken into account. For each comparison, genes were ranked based on their t‐value that was calculated by the moderated t‐test. Statistical significance of GSEA results was determined using 10.000 permutations.

## Results

### Presence of different cell types in IEC layers

The cellular makeup of the IEC layers was characterized by evaluating the expression of known cell type specific gene markers in the control samples. This was done to determine the similarities in cellular makeup between the IEC layers and the in vivo gut. Table 1 summarizes the expression of these marker genes and indicated that enterocytes, goblet cells, paneth cells, tuft cells, enteroendocrine cells, and stem cells were present in our IEC layers. In the total RNA pool, 13 out of 14 goblet cell markers were expressed, 13 out of 14 enterocyte (proximal) cell markers, 13 out of 14 enterocyte (distal) cell markers, 8 out of 14 paneth cell markers, 12 out of 14 tuft cell markers, 8 out of 14 enteroendocrine cell markers and 11 out of 14 stem cell markers.

**Table 1:**
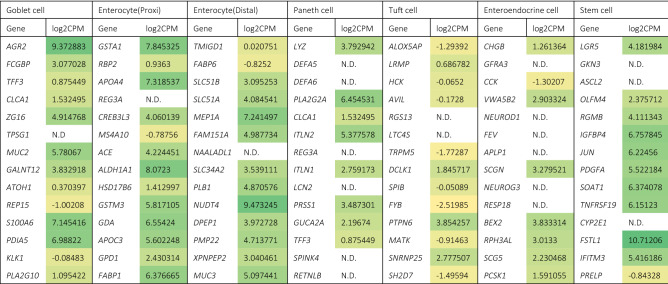
Gene expression in log2 values (counts per million) of selected marker genes for specific intestinal cell types in induced pluripotent stem cell derived intestinal epithelial cell layer control samples.

N.D.: not detectable. Colours were scaled from low expression (yellow) to high expression (green). Data are expressed as averages of 3 samples.

### Differential gene expression

**Figure 1:**
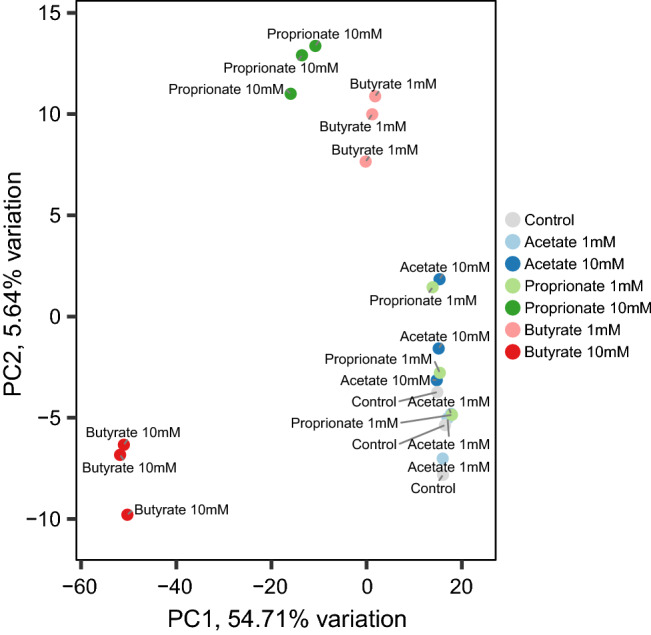
PCA plot of induced pluripotent stem cell derived intestinal epithelial cell layers exposed to 1 or 10 mM butyrate, propionate, acetate or control for 24 h.

Gene expression profiles of IEC layers exposed to the three individual SCFAs at two concentrations were evaluated by a PCA. A PCA scatterplot representing the first two principle components based on the transcriptome profiles of the six exposure groups and the controls is shown in Fig. 1. PC1 and PC2 explain 54.71% and 5.64% of the total variation, respectively. The control samples and samples exposed to 1 mM and 10 mM acetate, and 1 mM propionate clustered together, indicating little variation in gene expression between the groups. Based on the first component (PC1), samples exposed to 10 mM butyrate differed most from the control samples, as shown by the largest distance between the two groups. Also, samples exposed to 10 mM propionate and to a lesser extent samples exposed to 1 mM butyrate differed from the control samples and these groups were located between the control and butyrate 10 mM samples. Based on the second component (PC2), samples exposed to 10 mM propionate and 1 mM butyrate differed most from the control samples.

### Gene expression data analysis at the individual gene level

**Figure 2:**
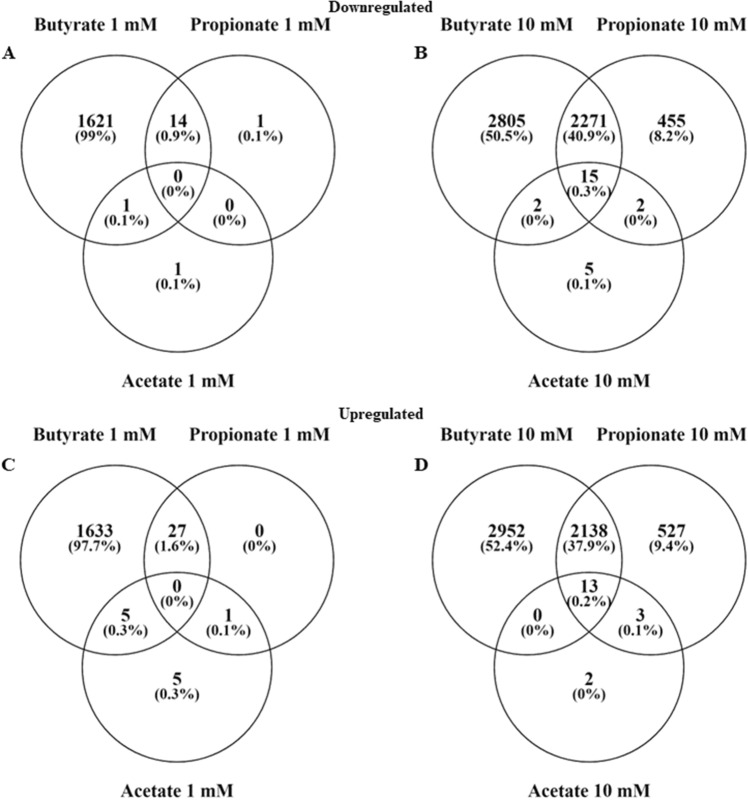
Venn diagrams showing the differentially expressed genes (FDR < 0.05) in induced pluripotent stem cell derived intestinal epithelial cell layers exposed to 1 or 10 mM butyrate, propionate or acetate for 24 h versus the control. (**A**) downregulated genes in IEC layers exposed to 1 mM butyrate, propionate or acetate, (**B**) downregulated genes in IEC layers exposed to 10 mM butyrate, propionate or acetate, (**C**) upregulated genes in IEC layers exposed to 1 mM butyrate, propionate or acetate, (**D**) upregulated genes in IEC layers exposed to 10 mM butyrate, propionate or acetate.

**Figure 3:**
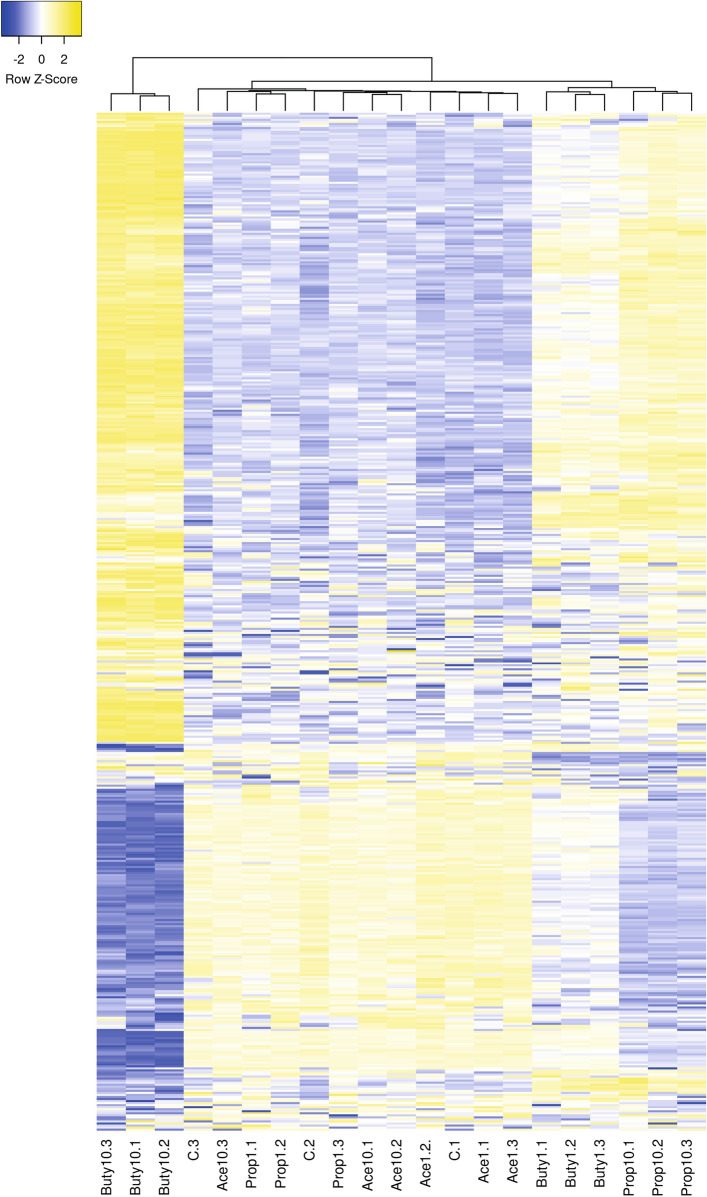
Heatmap of the top 500 differentially expressed genes in induced pluripotent stem cell derived intestinal epithelial cell layers exposed to 1 or 10 mM butyrate (Buty), propionate (Prop) or acetate (Ace) and in the control samples (C) with 3 samples per condition. Blue indicates below average expression and yellow indicates above average expression. Made using heatmapper, http://www.heatmapper.ca^43,49^.

The original dataset consisted of 60,237 genes in 21 samples, after filtering for low count genes this was reduced to 19,654 genes. Of these genes, 3,534 were not protein coding resulting in a data set containing 16,120 genes. For comparison of exposure conditions a False Discovery Rate (FDR) cut-off value of < 0.05 was used resulting in 1,636 down- and 1,665 upregulated genes for butyrate 1 mM, 5,093 down- and 5,103 upregulated genes for butyrate 10 mM, 15 down- and 28 upregulated genes for propionate 1 mM, 2,741 down- and 2,681 upregulated genes for propionate 10 mM, 2 down- and 11 upregulated genes for acetate 1 mM, and 24 down- and 18 upregulated genes for acetate 10 mM. At 1 mM there were no shared genes between all three compounds, at 10 mM there were 15 down- and 13 upregulated shared genes. Between butyrate and propionate there were 14 down- and 27- upregulated shared genes at 1 mM and 2,271 down- and 2,138 upregulated shared genes at 10 mM. Between butyrate and acetate there were 1 down- and 5- upregulated shared genes at 1 mM and 2 down- and 0 upregulated shared genes at 10 mM. Between acetate and propionate there was only 1 shared upregulated gene at 1 mM and 2 down- and 4 upregulated genes at 10 mM (Fig. 2). To obtain a visual overview of differential gene expression at the individual gene level all samples were hierarchically clustered based on the top 500 highest differentially expressed genes and presented in a heatmap (Fig. 3).

### SCFAs effects on IEC differentiation

To evaluate the effects of the SCFAs on differentiation of IECs, gene expression of specific differentiation and cell type markers was analysed in all exposure groups versus the control group. Table 2 summarizes the expression of these marker genes. The stem cell marker *LGR5* was significantly reduced after acetate 1 and 10 mM, propionate 10 mM and butyrate 10 mM exposure. The enterocyte marker Alkaline Phosphatase intestinal (*ALPi*) was significantly increased after acetate 1 mM and butyrate 1 mM exposure, but reduced after butyrate 10 mM exposure. The goblet cell marker Mucin 2 (*MUC2*) was significantly increased after exposure of the cells to 1 mM acetate, but significantly reduced after propionate 10 mM and butyrate 10 mM exposure. The enteroendocrine marker chromogranin A (*CHGA*) was significantly increased after butyrate 10 mM exposure. The Paneth cell marker lysozyme (*LYZ*) was significantly increased after acetate 1 mM exposure, but significantly reduced after butyrate 10 mM exposure. The tight junction marker claudin-3 (CLDN3) was significantly increased after butyrate 1 mM exposure, but significantly reduced after butyrate 10 mM exposure. Another tight junction marker, occludin (*OCLN*), was also significantly reduced after butyrate 10 mM exposure. A third tight junction marker, zona-occludens 1 (*TJP1*), was significantly reduced after acetate 10 mM, propionate 1 mM and 10 mM and butyrate 1 mM and 10 mM exposure. Finally, the tuft cell marker Doublecortin Like Kinase 1 (*DCLK1*) was significantly increased after butyrate 10 mM exposure. In general, exposure to butyrate 10 mM had most pronounced effects on the selected differentiation markers.

**Table 2:**
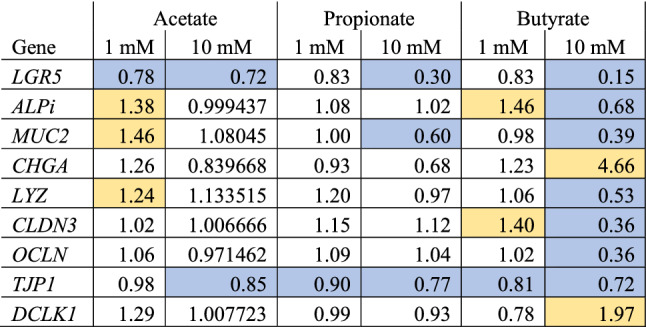
Gene expression data of specific differentiation and cell type markers.

Gene expression ratios in induced pluripotent stem cell derived intestinal epithelial cell layers exposed to 1 or 10 mM butyrate, propionate or acetate for 24 h compared with the control. Each ratio is based on the average expression data of 3 samples. Blue cells are significantly downregulated and yellow cells are significantly upregulated (*P* < 0.05). The markers represent stem cells (LGR5), enterocytes (Alkaline Phosphatase intestinal; ALPi), goblet cells (Mucin 2; Muc2), enteroendocrine cells (chromogranin A; CHGA), paneth cells (Lysozyme; LYZ), tight junction proteins (Claudin-3; CLDN3, Occludin; OCLN and Zonule-occludens 1; TJP1), and tuft cells (Doublecortin Like Kinase 1; DLK1).

### Gene set enrichment analysis of gene expression data from iPSC derived IECs exposed to 1 and 10 mM butyrate or 10 mM propionate

**Figure 4:**
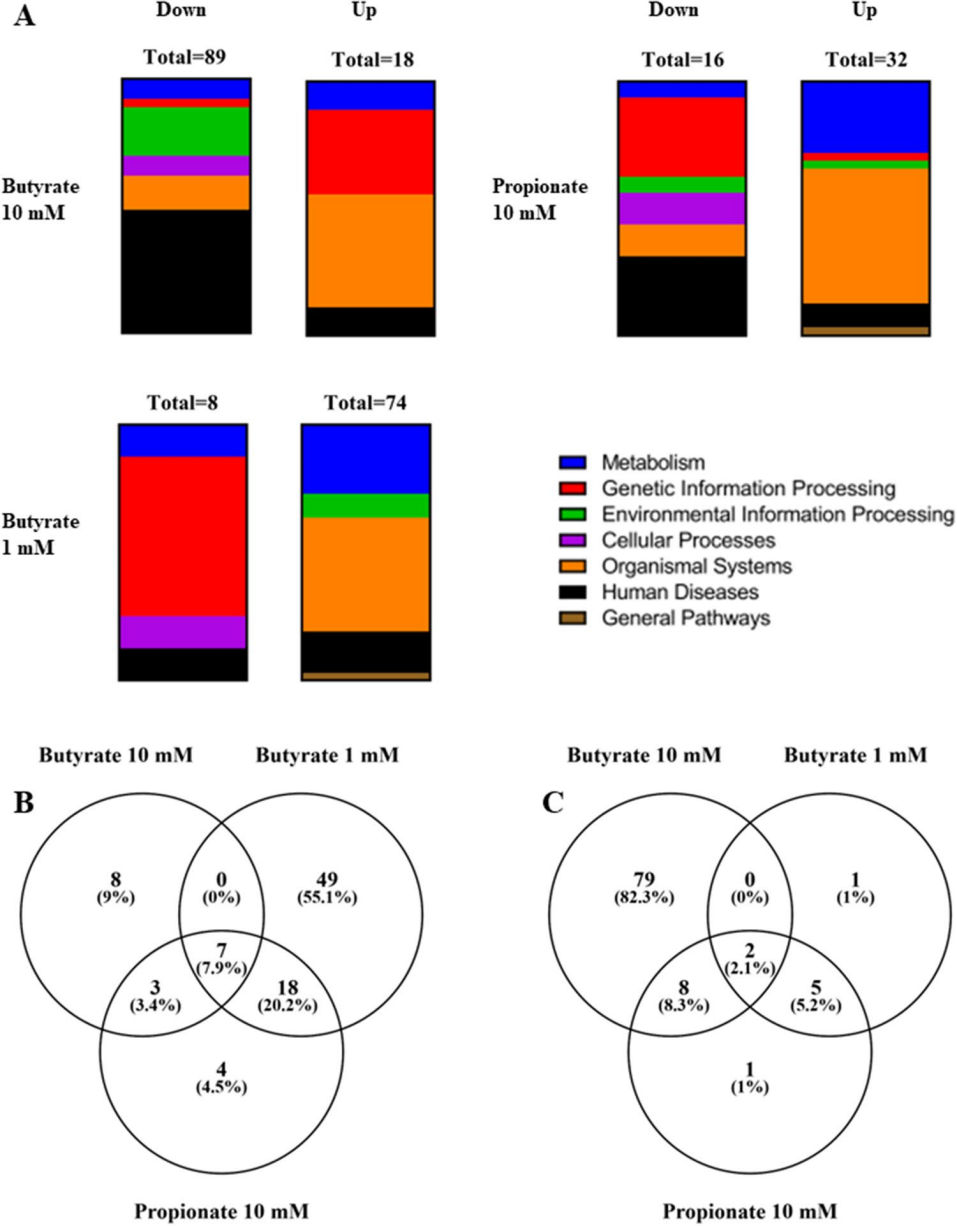
Differentially regulated pathways (*P* < 0.05, FDR < 0.25) in induced pluripotent stem cell derived intestinal epithelial cell layers exposed to 1 or 10 mM butyrate or 10 mM Propionate for 24 h. (**A**) vertical slice graphs showing the distribution of the up- or downregulated pathways over six KEGG categories. (**B**) Venn diagram showing the upregulated pathways, and (**C**) Venn diagram showing the downregulated pathways versus the control.

GSEA was performed for an in-depth evaluation of the effects of exposure to SCFAs on a biological pathway level. Six KEGG categories were included in the analysis; metabolism, genetic information processing, environmental information processing, cellular processes, organismal system and human diseases (BRITE Functional Hierarchy level 1). Gene sets were ranked based on the Normalised Enrichment Score (NES) to select the most enriched pathways. Cut-off values *P* < 0.05 and FDR < 0.25 were applied to select statistically significant pathways based on standard GSEA practice. Propionate at 1 mM exposure resulted in a total of 56 upregulated gene sets and a total of 0 downregulated gene sets. Acetate exposure at both 1 mM and 10 mM, resulted in a total of 95 and 2 upregulated gene sets and a total of 13 and 98 downregulated gene sets, respectively. Due to these relatively low numbers of enriched pathways and because they grouped together with the controls in previous analyses, propionate at 1 mM and acetate at both concentrations were excluded from this analysis, leaving the exposure groups butyrate 1 and 10 mM, and propionate 10 mM. Exposure to butyrate 1 mM resulted in a total of 8 downregulated gene sets divided over the metabolism, genetic information processing, cellular processes and human diseases categories and 74 upregulated gene sets divided over the metabolism, environmental information processing, organismal systems, human diseases and general categories (Fig. 4A). Butyrate 10 mM exposure resulted in a total of 89 downregulated gene sets divided over all 6 categories (Metabolism, Genetic Information Processing, Environmental Information Processing, Cellular Processes, Organismal Systems and Human Diseases) and 18 upregulated gene sets divided over the metabolism, genetic information processing, organismal systems, and human diseases categories (Fig. 4A). Propionate 10 mM exposure resulted in a total of 16 downregulated gene sets divided over all 6 categories and 32 upregulated gene sets which were also divided over all 6 categories (Fig. 4A). When comparing the three exposure groups there were 7 shared upregulated gene sets and 2 shared downregulated gene sets for all three exposures. There were no additional shared gene sets between the butyrate 10 and 1 mM exposure groups. The butyrate 10 mM and propionate 10 mM exposure groups shared 3 upregulated and 8 downregulated gene sets, while the butyrate 1 mM and propionate 10 mM exposure groups shared 18 upregulated and 5 downregulated gene sets (Fig. 4B, C).

For further analysis on the pathway level, pathways related to the intestine were selected based on expert judgement. The top 10 of this selection, is given in Tables 3, 4, 5 for butyrate, propionate and acetate exposure induced pathways, respectively. Following butyrate 10 mM exposure the most upregulated gene set was DNA replication in the KEGG category Genetic Information Processing and the KEGG category subgroup Replication and repair (NES = 2.43), the most downregulated gene set was the p53 signalling pathway in the KEGG category Human Diseases and the KEGG category subgroup Cell growth and death (NES = − 1.99) (Table 3). Following butyrate 1 mM exposure the most upregulated gene set was chemical carcinogenesis in the KEGG category Human Diseases and KEGG category subgroup Cancer: overview (NES = 2.26), the most downregulated gene set was the proteasome set in the KEGG category Genetic Information Processing and the KEGG category subgroup Folding, sorting and degradation (NES = − 2.17) (Table 4). Following propionate 10 mM exposure the most upregulated gene set was circadian entrainment in the KEGG category Organismal Systems and KEGG category subgroup Environmental adaptation (NES = 1.83), the most downregulated gene set was the p53 signalling pathway in the KEGG category Human Diseases and the KEGG category subgroup Cell growth and death (NES = − 2.08) (Table 5).

**Table 3:** Top 10 most enriched (based on NES) up- and downregulated pathways related to the intestine in induced pluripotent stem cell derived intestinal epithelial cell layers exposed to 10 mM butyrate compared with the control.

KEGG pathway name	KEGG category	KEGG category subgroup	Genes	NES	*P*-value	FDR
**Upregulated pathways**^**a**^						
DNA replication	Genetic Information Processing	Replication and repair	36	2.43	0.000	0.000
Mismatch repair	Genetic Information Processing	Replication and repair	23	2.18	0.000	0.000
Fanconi anemia pathway	Genetic Information Processing	Replication and repair	54	2.15	0.000	0.000
Homologous recombination	Genetic Information Processing	Replication and repair	41	1.99	0.000	0.004
RNA polymerase	Genetic Information Processing	Transcription	29	1.72	0.007	0.058
Fatty acid biosynthesis	Metabolism	Lipid metabolism	16	1.59	0.027	0.142
Nucleotide excision repair	Genetic Information Processing	Replication and repair	46	1.59	0.012	0.128
Endocrine and other factor regulated calcium reabsorption	Organismal Systems	Excretory system	48	1.55	0.012	0.135
Alanine, aspartate and glutamate metabolism	Metabolism	Amino acid metabolism	35	1.48	0.035	0.150
Mineral absorption	Organismal Systems	Digestive system	56	1.47	0.022	0.143
**Downregulated pathways**^**a**^						
P53 signaling pathway	Cellular Processes	Cell growth and death	73	− 1.99	0.000	0.010
Pathogenic Escherichia Coli infection	Human Diseases	Infectious disease: bacterial	179	− 1.97	0.000	0.007
Steroid hormone biosynthesis	Metabolism	Lipid metabolism	49	− 1.95	0.000	0.007
Shigellosis	Human Diseases	Infectious disease: bacterial	224	− 1.91	0.000	0.010
ECM receptor interaction	Environmental Information Processing	Signaling molecules and interaction	78	− 1.86	0.000	0.013
NF kappa B signaling pathway	Environmental Information Processing	Signal transduction	94	− 1.83	0.000	0.020
Glutathione metabolism	Metabolism	Metabolism of other amino acids	46	− 1.81	0.001	0.023
Hippo signaling pathway multiple species	Environmental Information Processing	Signal transduction	29	− 1.80	0.003	0.019
Regulation of actin cytoskeleton	Cellular Processes	Cell motility	194	− 1.74	0.000	0.030
JAK STAT signaling pathway	Environmental Information Processing	Signal transduction	110	− 1.73	0.000	0.030

^a^References indicating their role or relationship to the intestine for the selected pathways are given in the supplementary files; Table S1.

**Table 4:** Top 10 most enriched (based on NES) up- and downregulated pathways related to the intestine in induced pluripotent stem cell derived intestinal epithelial cell layers exposed to 1 mM butyrate compared with the control.

KEGG pathway name	KEGG category	KEGG category subgroup	Genes	NES	*P*-value	FDR
**Upregulated pathways**^**a**^						
Chemical carcinogenesis	Human Diseases	Cancer: overview	65	2.26	0.000	0.000
Mineral absorption	Organismal Systems	Digestive system	56	2.26	0.000	0.000
Fat digestion and absorption	Organismal Systems	Digestive system	30	2.21	0.000	0.000
Tryptophan metabolism	Metabolism	Amino acid metabolism	36	2.02	0.000	0.003
Glycine serine and threonine metabolism	Metabolism	Amino acid metabolism	37	1.97	0.000	0.004
Circadian entrainment	Organismal Systems	Environmental adaptation	86	1.86	0.000	0.018
Metabolism of xenobiotics by cytochrome P450	Metabolism	Xenobiotics biodegradation and metabolism	57	1.86	0.001	0.015
Ubiquinone and other terpenoid quinone biosynthesis	Metabolism	Metabolism of cofactors and vitamins	11	1.86	0.001	0.014
PPAR signaling pathway	Organismal Systems	Endocrine system	69	1.85	0.000	0.014
Arachidonic acid metabolism	Metabolism	Lipid metabolism	47	1.81	0.002	0.017
**Downregulated pathways**^**a**^						
Proteasome	Genetic Information Processing	Folding, sorting and degradation	43	− 2.17	0.000	0.001
P53 signaling pathway	Cellular Processes	Cell growth and death	73	− 2.16	0.000	0.000
Spliceosome	Genetic Information Processing	Transcription	134	− 1.99	0.000	0.004
Ribosome biogenesis in eukaryotes	Genetic Information Processing	Translation	73	− 1.93	0.000	0.007
RNA transport	Genetic Information Processing	Translation	157	− 1.91	0.000	0.007
Starch and sucrose metabolism	Metabolism	Carbohydrate metabolism	28	− 1.68	0.010	0.073
MRNA surveillance pathway	Genetic Information Processing	Translation	90	− 1.60	0.002	0.130

^a^References indicating their role or relationship to the intestine for the selected pathways are given in the supplementary files; Table S2.

**Table 5:** Top 10 most enriched (based on NES) up- and downregulated pathways related to the intestine in induced pluripotent stem cell derived intestinal epithelial cell layers exposed to 10 mM propionate compared with the control.

KEGG pathway name	KEGG category	KEGG category subgroup	Genes	NES	*P*-value	FDR
**Upregulated pathways**^**a**^						
Circadian entrainment	Organismal Systems	Environmental adaptation	86	1.83	0.000	0.059
Thiamine metabolism	Metabolism	Metabolism of cofactors and vitamins	15	1.82	0.002	0.046
Mismatch repair	Genetic Information Processing	Replication and repair	23	1.77	0.005	0.058
Tryptophan metabolism	Metabolism	Amino acid metabolism	36	1.76	0.002	0.045
Fat digestion and absorption	Organismal Systems	Digestive system	30	1.74	0.003	0.046
Glycine serine and threonine metabolism	Metabolism	Amino acid metabolism	37	1.68	0.008	0.060
Aldosterone regulated sodium reabsorption	Organismal Systems	Excretory system	33	1.65	0.010	0.065
Endocrine and other factor regulated sodium reabsorption	Organismal Systems	Excretory system	48	1.64	0.008	0.073
Longevity regulating pathway	Organismal Systems	Aging	59	1.60	0.007	0.085
Mineral absorption	Organismal Systems	Digestive system	56	1.58	0.008	0.092
**Downregulated pathways**^**a**^						
P53 signaling pathway	Cellular Processes	Cell growth and death	73	− 2.08	0.000	0.003
Proteasome	Genetic Information Processing	Folding, sorting and degradation	43	− 2.00	0.000	0.006
NF kappa B signaling pathway	Environmental Information Processing	Signal transduction	94	− 1.93	0.000	0.011
Spliceosome	Genetic Information Processing	Transcription	134	− 1.86	0.000	0.021
Pathogenic Escherichia Coli infection	Human Diseases	Infectious disease: bacterial	179	− 1.84	0.000	0.020
Ribosome biogenesis in eukaryotes	Genetic Information Processing	Translation	73	− 1.82	0.000	0.024
RNA transport	Genetic Information Processing	Translation	157	− 1.79	0.000	0.029
Shigellosis	Human Diseases	Infectious disease: bacterial	224	− 1.75	0.000	0.035
Protein processing in endoplasmic reticulum	Genetic Information Processing	Folding, sorting and degradation	163	− 1.66	0.001	0.085
Starch and sucrose metabolism	Metabolism	Carbohydrate metabolism	28	− 1.59	0.021	0.133

^a^References indicating their role or relationship to the intestine for the selected pathways are given in the supplementary files; Table S3.

## Discussion

The aim of the current study was to investigate the effects of three short chain fatty acids on gene expression profiles of human induced pluripotent stem cell (iPSC) derived intestinal epithelial cell (IEC) layers by whole genome analysis. The IEC layers were exposed to acetate, propionate and butyrate at 1 and 10 mM for 24 h. Analysis of RNA-sequencing data revealed differential gene expression of 16,120 unique protein coding genes when comparing the treatments to their controls, where exposure to butyrate resulted in the strongest responses, followed by propionate, while acetate only marginally affected the gene expression profile of the IEC layers.

Human iPSCs that are differentiated into IEC layers have previously been shown to differentiate into multiple cell types (i.e. enterocytes, goblet cells, Paneth cells, tuft cells, and enteroendocrine cells) that are commonly present in the human intestinal epithelium in vivo^50–53^. To confirm the presence of these cell types in our IEC model we evaluated the expression of a panel of cell specific marker genes. We selected 14 marker genes for each important intestinal cell type by searching literature and KEGG cell type signature sets^44,45^ (Table 1). Enterocytes are the most abundantly present cell type in the intestinal epithelium (in vivo), which were divided into proximal (i.e. duodenum/jejenum) and distal (i.e. ileum) enterocytes. Of the marker gene sets for both the proximal and distal enterocytes, 13 of the marker genes were expressed, indicating that the model is not specific for one of these two regions. The IEC layers clearly contained enterocytes with a small intestinal gene expression footprint, but due to a lack of markers specific for colonic cells we cannot confirm that the model only contains small intestinal cells. Similarly, expression of 13 markers for goblet cells was observed, indicating the presence of goblet cells in the IEC layers. For Paneth and enteroendocrine cells, expression of several of the marker genes was not detectable. Each of the cell types makes up for < 5% of the total amount of cells in the intestinal epithelium in vivo^44,54,55^. Therefore, a low signal may be expected as pooled RNA from all IECs was analyzed. The expression of tuft cell markers in our IEC layer was the lowest, which again is likely due to a low abundance of the cells in the IEC layers. Literature reported a very low abundance of tuft cells in the human intestine and in adult mouse intestinal epithelium they only represented 0.4% of the total amount of cells^45,56^. Lastly, we also observed expression of gene markers for stem cells, most importantly the well-known marker *LGR5*, which had high expression values indicating an abundance of stem cells as observed in mammalian crypts in vivo^57^. In summary, based on gene expression evaluation, the human iPSC derived IEC layers used in this study contained all important cell types found in the human intestine thus representing the cellular complexity as seen in vivo. However, based on the present data we were not able to identify whether the iPSC derived IECs are more resembling the small or large intestine.

The used exposure concentrations of butyrate, propionate and acetate (1 mM and 10 mM) were based on a recent study in adult stem cell derived IECs^32^, and selected to represent human in vivo intestinal concentrations. Regional differences in SCFA concentrations in the human intestine have been reported. Total SCFA concentrations typically range from 50 to 100 mM in the colonic lumen depending on the diet, whereas in the (distal) small intestine the concentrations are lower at around 11-13 mM^3^. Higher concentrations of 98.2 mM acetate, 3.4 mM propionate and 17.4 mM butyrate have also been reported in the small intestine, but these were measured in ileostoma patients and the increased concentration was likely due to the high microbial activity in the small intestine in these patients^58^. Acetate represents approximately 60% of the total concentration of SCFAs in the intestine with butyrate and propionate each constituting ~ 20%^3^. This means that the used concentrations of butyrate and propionate in the present study were relatively close to in vivo concentrations in both parts of the gut. For acetate, the highest concentration that was used was within the small intestine concentration range, but was below the concentration range reported for the colon. This may explain the low number of differentially expressed genes following acetate exposure. Alternatively human IECs might be less sensitive to exogenous acetate exposure as they have been described to produce acetate themselves where intracellular concentrations can reach micromolar concentrations, although this mostly happens in nutrient-deprived situations^59^. The timepoint of 24 h was chosen as SCFAs are chronically exposed in the human intestine, there will likely be different effects at earlier timepoints but these will be less relevant for comparison to the in vivo situation.

Exposure to butyrate, propionate and acetate at two concentrations resulted in varying numbers of genes with acetate only inducing a low number of genes compared to butyrate and propionate. This corroborates previously reported observations in IEC layers derived from adult human (and mouse) stem cells, where acetate at 1 and 10 mM also induced differential gene expression to a lesser extent than propionate and butyrate at 1 mM and 10 mM, albeit only a select number of genes was examined^32^.It is known that SCFAs have an effect on cell differentiation^32,60,61^. Therefore, the effects of the SCFAs on the expression of selected marker genes for specific intestinal cell types and on marker genes related to intestinal cell differentiation were evaluated. Again, propionate 10 mM, butyrate 1 mM and butyrate 10 mM exposure had the most pronounced effects on the selected marker genes when compared with the control samples (Table 2). It is widely known that butyrate reduces the expression of LGR5 in adult stem cell derived human intestinal organoids, which is a marker gene for stem cells^32,62^, thereby indicating that differentiation of the cells is induced. In the present study, LGR5 expression is also reduced after exposure to butyrate 10 mM as were most of the other selected cell markers. The acetate 10 mM, propionate 1 and 10 mM exposure groups showed less pronounced effects on differentiation markers, but in general the selected markers were mostly downregulated upon exposure. However, exposure to butyrate 1 mM and acetate 1 mM resulted in more upregulated than downregulated marker genes. When comparing the gene expression data of the selected marker genes from the current study with those reported in literature in adult stem cell derived organoids exposed to the same SCFAs under the same conditions^32^, marked differences in gene expression were observed, which were most prominent after butyrate 10 mM exposure. In this exposure group 4 out of the 9 studied genes (i.e. ALPi, LYZ, CLDN3 and TJP1) were downregulated in the present study versus upregulated in the study by Pearce et al. (2020). Furthermore, the IEC layers in the present study appeared to be less sensitive to acetate 10 mM exposure compared with the adult stem cell derived organoids as expression of most genes remained unchanged while they were generally upregulated in the adult stem cell derived organoids (Pearce et al., 2020). In contrast, acetate 1 mM exposure induced expression of several of the marker genes in the present study, which remained unchanged in the adult stem cell derived organoids (Pearce et al., 2020). The results show that gene expression, and the potential subsequent effects on cell differentiation, is highly concentration-dependent, but also that the responses are model dependent. Variability in gene expression profiles between different cell models is well-known for cell-line based models, and it appears to be true as well for (adult or induced pluripotent) stem cell derived IEC models. Likely explanations are the differences in origin of the stem cells (human iPSC versus human adult stem cells), differences between donors and the methodology that is used to differentiate the stem cells, which is often optimised for specific cell marker expression^51–53^.

Both the PCA analysis and the hierarchical clustering indicated a distinct effect on gene expression profiles following exposure to 10 mM butyrate compared with all other exposures, while the gene expression profiles following butyrate 1 mM and propionate 10 mM clustered together in both the PCA and the hierarchical clustering. The remaining exposure conditions (i.e. 1 and 10 mM acetate, 1 mM propionate) clustered together with the control exposure group in both the PCA and the hierarchical clustering indicating little effect of the exposure. Pathway analysis (i.e. GSEA) on the data from the exposure groups with a high number of differentially expressed genes (i.e. propionate 10 mM, butyrate 1 mM and butyrate 10 mM) revealed a clear concentration-dependent switch in the differential gene expression in the butyrate exposure groups from mostly upregulated pathways at 1 mM (8 down- and 72 upregulated pathways) to mostly downregulated pathways at 10 mM (89 down- and 18 upregulated pathways). Exposure to the low butyrate concentration appeared to induce metabolism and absorption processes as indicated by the upregulated pathways (7 pathways out of the top 10). Pathways related to genetic information processing were mainly downregulated (5 pathways out of the top 7) and were related to RNA transcription, suggesting that low butyrate concentrations could lead to a downregulation of protein synthesis. Six of the top 10 induced pathways, following exposure to 10 mM butyrate, were related to genetic information processing and could be connected to DNA replication and repair, suggesting that high concentrations can induce DNA damage, but there were no indications of induced apoptosis or cell death. Four of the top 10 downregulated pathways, following exposure to butyrate 10 mM, were related to signal transduction. Propionate 10 mM had overall less affected pathways in comparison with butyrate exposure, 47 pathways in total, and the numbers were less skewed with 16 down- and 31 upregulated pathways. It appears that propionate 10 mM exposure induced absorption and metabolism processes as 7 of the top 10 upregulated pathways were connected to these processes. In the downregulated pathways 5 out of 10 pathways were in the genetic information processing category and related mostly to RNA and protein processing processes. Interestingly, many of the up- and downregulated pathways in the top 10 were similar to those of the butyrate 1 mM exposure, indicating that these treatments seem to have similar effects.

Upregulation of DNA replication and repair pathways, as mainly seen after butyrate 10 mM exposure, indicates DNA damage, which in turn is associated with HDAC inhibition. Indeed butyrate has been described to induce HDAC inhibition^63,64^, corroborating our results after exposure to 10 mM butyrate. This effect appears to be both concentration-dependent and SCFA dependent, as we do not see similarity in upregulated pathways after butyrate 1 mM or propionate 10 mM exposure. Propionate has been described to have a lower HDAC inhibitory capacity than butyrate^65^, again corroborating the observations in the current study. Another common finding in literature is that butyrate decreases cell proliferation, which was not observed in the present study. However, this effect is reported from studies that used cancer cells like Caco-2 or HT-29^9,19,60,66^. As mentioned earlier, these cell-lines are not the most suitable systems to study butyrate effects as cancer cells do not use butyrate as their energy source, which leads to an intracellular build-up of butyrate and eventually leads to cell death. Therefore, human IEC and cell-line model differences in energy homeostasis and thus proliferation may be expected.

A strong downregulation of the p53 pathway was seen in the butyrate 1 mM, 10 mM and propionate 10 mM exposure groups. The p53 pathway is induced in response to stress factors that affect DNA replication and induces apoptosis and senescence^67,68^. DNA replication pathways were clearly affected after butyrate 10 mM exposure, but there were no signs of induced apoptosis, which could be due to this strong reduction of the p53 pathway. In contrast, in cancer cell lines both p53 dependent and independent apoptosis pathways have been reported to be induced after exposure to butyrate^18,69–71^, but again the large differences between model systems, and the build-up of butyrate in cancer cell lines, could explain these differences in effects.

Some pathways in the category human diseases were strongly regulated after exposure to butyrate 1 mM and 10 mM, and propionate 10 mM, indicating that the SCFAs appear to induce responses that have been linked to certain diseases. Examination of the underlying mechanisms of these disease pathways gives insights in the effects of the SCFAs. The butyrate 10 mM and propionate 10 mM exposure groups downregulated a number of bacterial disease pathways including *E. coli* and *Shigella* infections. Bacterial infections are known to induce inflammation or other immune responses and inspection of the downregulated pathways indeed showed many pathways connected to immune responses that were downregulated, especially after butyrate 10 mM exposure. Commensal bacteria that are known to produce SCFAs are reported to reduce inflammatory intestinal reactions^6,12,72^. In the butyrate 1 mM exposure group, the chemical carcinogenesis pathway was the most upregulated pathway. This is a very general pathway including genes of the CYP family, glutathione transferases and sulfotransferases, the observed upregulation is most likely related to butyrate effects on CYP enzymes in relation to metabolism^73–76^, which is corroborated by the upregulated metabolism of xenobiotics by cytochrome P450 pathway in the butyrate 1 mM exposure group.

Both butyrate and propionate induced many different types of metabolism related pathways, with amino acid metabolism pathways being regulated the most. Both butyrate and propionate are used as an energy source by intestinal epithelial cells^77,78^, which is confirmed by the upregulated fatty acid metabolism biosynthesis pathway after butyrate 10 mM exposure. This pathway encompasses the metabolism of butyrate and propionate. The alanine, aspartate and glutamate metabolism pathway was also upregulated after butyrate 10 mM exposure. This pathway is very important in energy household and the ability to use dietary amino acids for biosynthesis^79^ and it was previously linked to butyrate metabolism via L-glutamate^80^. Lastly, a downregulation of the starch and sucrose metabolism pathway following exposure to butyrate 1 mM and propionate 10 mM was observed. The end product of this pathway, glucose, is one of the main energy sources for cells^81,82^. This is extremely interesting as a lower dependency of the IEC layers on this pathway suggests that they can switch to SCFAs as an energy source, something that is described not to occur in cancer cell lines like Caco-2 and is considered a major disadvantage of cancer cell lines for SCFA studies.

Reviews on the effects of SCFAs on the human intestine report a wide variety of effects^2,83^ where traditional models were unable to model these due to the butyrate paradox, comparatively the IPSC derived IEC combined with RNA-seq has been able to capture a wide variety of effects pointing toward this model being a more suitable in vitro cell model for SCFA studies when compared with cancer cell lines. This also implies that this IEC model may be better suited for development of more complex in vitro intestinal models combining microbes with intestinal cells in for example gut-on-a-chip devices^84–86^. When building further on this type of model there are other things to consider as well, like how compounds are exposed to the cells. Results from this study and literature have shown a reduction of LGR5 expression upon SCFA exposure indicating a reduction of stem cells. When aiming at developing a cell model for long term studies, a pool of stem cells would be required. In vivo, stem cells are protected from exposure by their location in the crypts, which could be modelled in vitro for example by recreating the crypts using 3D scaffolds^62,83,87^.

## Conclusion

The aim of this study was to evaluate the effects of the SCFAs butyrate, propionate and acetate at two concentrations in human iPSC derived IEC layers using RNA-seq analysis. This novel model is a biologically more relevant cell model in comparison to cancer cell lines, but little is known about its performance in relation to SCFA exposure. Transcriptomics analysis revealed unique gene expression profiles for the three SCFAs, with expressions profiles that were also dose-dependent. Exposure to butyrate resulted in the strongest responses, followed by propionate, while acetate only marginally affected the gene expression profiles of the IECs. Several known effects of SCFAs on intestinal cells were confirmed, such as effects on metabolism and immune responses. The changes in metabolic pathways in the intestinal epithelial cell layers in this study demonstrate that there is a switch in energy homeostasis, this is likely associated with the use of SCFAs as an energy source by the induced pluripotent stem cell derived intestinal epithelial cells similar to in vivo intestinal tissues where butyrate is an important energy source.

## Supplementary Information

Supplementary Information.

## Data Availability

The data that support the findings of this study will be openly available in GEO DataSets (GSE200309, https://www.ncbi.nlm.nih.gov/geo/query/acc.cgi?acc=GSE200309).
