# Supplementary material for: Differential gene expression in iPSC-derived human intestinal epithelial cell layers following exposure to two concentrations of butyrate, propionate and acetate
Source: Sci Rep. 2022 Aug 17;12:13988. doi: 10.1038/s41598-022-17296-8 (PMC9385623; doi:10.1038/s41598-022-17296-8)
Supplement: Supplementary file 1 — Supplementary Information. [file 41598_2022_17296_MOESM1_ESM.docx]

**Supplementary file belonging to:**

**Differential gene expression in iPSC derived human intestinal epithelial cell layers following exposure to two concentrations of the Short Chain Fatty Acids butyrate, propionate and acetate**

Menno Grouls^1^, Aafke W.F. Janssen^2^, Loes P.M. Duivenvoorde^2^, Guido J.E.J. Hooiveld^3^, Hans Bouwmeester^1^, and Meike van der Zande^2^

^1^ Division of Toxicology, Wageningen University, Wageningen University & Research, Postbus 8000, 6700 EA, Wageningen, The Netherlands

^2^ Wageningen Food Safety Research, Wageningen University & Research, Wageningen, The Netherlands

^3^ Division of Human Nutrition, Wageningen University, Wageningen University & Research, Wageningen, The Netherlands

Table S 1 butyrate 10 mM references indicating their role or relationship to the intestine

| KEGG pathway name | Reference |
| --- | --- |
| **Upregulated pathways** | |
| DNA replication | ^1^ |
| Mismatch repair | ^2,3^ |
| Fanconi anemia pathway | ^4,5^ |
| Homologous recombination | ^6,7^ |
| RNA polymerase | ^8^ |
| Fatty acid biosynthesis | ^9,10^ |
| Nucleotide excision repair | ^11^ |
| Endocrine and other factor regulated calcium reabsoprtion | ^12,13^ |
| Alanine, aspartate and glutamate metabolism | ^14^ |
| Mineral absorption | ^15–18^ |
| **Downregulated pathways** | |
| P53 signaling pathway | ^19^ |
| Pathogenic Escherichia Coli infection | ^20^ |
| Steroid hormone biosynthesis | ^21^ |
| Shigellosis | ^22,23^ |
| ECM receptor interaction | ^24^ |
| NF kappa B signaling pathway | ^25,26^ |
| Glutathione metabolism | ^27^ |
| Hippo signaling pathway multiple species | ^28^ |
| Regulation of actin cytoskeleton | ^29^ |
| JAK STAT siganling pathway | ^30,31^ |

Table S2 butyrate 1 mM references indicating their role or relationship to the intestine

| KEGG pathway name | References |
| --- | --- |
| Upregulated pathways | |
| Chemical carcinogenesis | ^32^ |
| Mineral absorption | ^15–18^ |
| Fat digestion and absorption | ^33^ |
| Tryptophan metabolism | ^34,35^ |
| Glycine serine and threonine metabolism | ^36,37^ |
| Circadian entrainment | ^38,39^ |
| Metabolism of xenobiotics by cytochrome P450 | ^40^ |
| Ubiquinone and other terpenoid quinone biosynthesis | ^41^ |
| PPAR signaling pathway | ^42,43^ |
| Arachidonic acid metabolism | ^44^ |
| Downregulated pathways | |
| Proteasome | ^45,46^ |
| P53 signaling pathway | ^19^ |
| Spliceosome | ^47^ |
| Ribosome biogenesis in eukaryotes | ^48,49^ |
| RNA transport | ^50^ |
| Starch and sucrose metabolism | ^51,52^ |
| MRNA surveillance pathway | ^53,54^ |

Table S3 propionate 10 mM references indicating their role or relationship to the intestine

| KEGG pathway name | References |
| --- | --- |
| Upregulated pathways | |
| Circadian entrainment | ^38,39^ |
| Thiamine metabolism | ^55,56^ |
| Mismatch repair | ^2,3^ |
| Tryptophan metabolism | ^34,35^ |
| Fat digestion and absorption | ^33^ |
| Glycine serine and threonine metabolism | ^36,37^ |
| Aldosterone regulated sodium reabsorption | ^57^ |
| Endocrine and other factor regulated sodium reabsorption | ^12,13^ |
| Longevity regulating pathway | ^58–60^ |
| Mineral absorption | ^15–18^ |
| Downregulated pathways | |
| P53 signaling pathway | ^19^ |
| Proteasome | ^45,46^ |
| NF kappa B signaling pathway | ^25,26^ |
| Spliceosome | ^47^ |
| Pathogenic Escherichia Coli infection | ^20^ |
| Ribosome biogenesis in eukaryotes | ^48,49^ |
| RNA transport | ^50^ |
| Shigellosis | ^22,23^ |
| Protein processing in endoplasmic reticulum | ^61,62^ |
| Starch and sucrose metabolism | ^51,52^ |

1. Waga S, Stillman B. The DNA replication fork in eukaryotic cells. *Annu Rev Biochem*. 1998;67:721-751. doi:10.1146/annurev.biochem.67.1.721

2. Li GM. Mechanisms and functions of DNA mismatch repair. *Cell Res*. 2008;18(1):85-98. doi:10.1038/cr.2007.115

3. Jiricny J. The multifaceted mismatch-repair system. *Nat Rev Mol Cell Biol*. 2006;7(5):335-346. doi:10.1038/nrm1907

4. Hays L, Frohnmayer D, Frohnmayer L, Larsen K, Owen J. Fanconi Anemia: Guidelines for Diagnosis and Management. *Fanconi Anemia Res Fund, Inc*. Published online June 3, 2014:1-391. Accessed June 22, 2021. http://www.ncbi.nlm.nih.gov/books/NBK1401/

5. Moldovan GL, D’Andrea AD. How the fanconi anemia pathway guards the genome. *Annu Rev Genet*. 2009;43:223-249. doi:10.1146/annurev-genet-102108-134222

6. Michel B, Boubakri H, Baharoglu Z, LeMasson M, Lestini R. Recombination proteins and rescue of arrested replication forks. *DNA Repair (Amst)*. 2007;6(7):967-980. doi:10.1016/j.dnarep.2007.02.016

7. Maloisel L, Fabre F, Gangloff S. DNA Polymerase δ Is Preferentially Recruited during Homologous Recombination To Promote Heteroduplex DNA Extension. *Mol Cell Biol*. 2008;28(4):1373-1382. doi:10.1128/mcb.01651-07

8. Cramer P, Armache KJ, Baumli S, et al. Structure of eukaryotic RNA polymerases. *Annu Rev Biophys*. 2008;37:337-352. doi:10.1146/annurev.biophys.37.032807.130008

9. Hiltunen JK, Chen Z, Haapalainen AM, Wierenga RK, Kastaniotis AJ. Mitochondrial fatty acid synthesis - An adopted set of enzymes making a pathway of major importance for the cellular metabolism. *Prog Lipid Res*. 2010;49(1):27-45. doi:10.1016/j.plipres.2009.08.001

10. Kastaniotis AJ, Autio KJ, Kerätär JM, et al. Mitochondrial fatty acid synthesis, fatty acids and mitochondrial physiology. *Biochim Biophys Acta - Mol Cell Biol Lipids*. 2017;1862(1):39-48. doi:10.1016/j.bbalip.2016.08.011

11. Friedberg EC. How nucleotide excision repair protects against cancer. *Nat Rev Cancer*. 2001;1(1):22-33. doi:10.1038/35094000

12. Van Abel M, Hoenderop JGJ, Van der Kemp AWCM, Van Leeuwen JPTM, Bindels RJM. Regulation of the epithelial Ca2+ channels in small intestine as studied by quantitative mRNA detection. *Am J Physiol - Gastrointest Liver Physiol*. 2003;285(1 48-1). doi:10.1152/ajpgi.00036.2003

13. Van Cromphaut SJ, Dewerchin M, Hoenderop JGJ, et al. Duodenal calcium absorption in vitamin D receptor-knockout mice: Functional and molecular aspects. *Proc Natl Acad Sci U S A*. 2001;98(23):13324-13329. doi:10.1073/pnas.231474698

14. Wu G. Intestinal mucosal amino acid catabolism. *J Nutr*. 1998;128(8):1249. doi:10.1093/jn/128.8.1249

15. Quamme GA. Recent developments in intestinal magnesium absorption. *Curr Opin Gastroenterol*. 2008;24(2):230-235. doi:10.1097/MOG.0b013e3282f37b59

16. Lichten LA, Cousins RJ. Mammalian zinc transporters: Nutritional and physiologic regulation. *Annu Rev Nutr*. 2009;29:153-176. doi:10.1146/annurev-nutr-033009-083312

17. Lönnerdal B. Intestinal regulation of copper homeostasis: A developmental perspective. In: *American Journal of Clinical Nutrition*. Vol 88. American Society for Nutrition; 2008:846S-850S. doi:10.1093/ajcn/88.3.846s

18. Dunn LL, Rahmanto YS, Richardson DR. Iron uptake and metabolism in the new millennium. *Trends Cell Biol*. 2007;17(2):93-100. doi:10.1016/j.tcb.2006.12.003

19. Harris SL, Levine AJ. The p53 pathway: Positive and negative feedback loops. *Oncogene*. 2005;24(17):2899-2908. doi:10.1038/sj.onc.1208615

20. Lai Y, Rosenshine I, Leong JM, Frankel G. Intimate host attachment: Enteropathogenic and enterohaemorrhagic Escherichia coli. *Cell Microbiol*. 2013;15(11):1796-1808. doi:10.1111/cmi.12179

21. Bouguen G, Dubuquoy L, Desreumaux P, Brunner T, Bertin B. Intestinal steroidogenesis. *Steroids*. 2015;103:64-71. doi:10.1016/j.steroids.2014.12.022

22. Schnupf P, Sansonetti PJ. Shigella Pathogenesis: New Insights through Advanced Methodologies . *Microbiol Spectr*. 2019;7(2). doi:10.1128/microbiolspec.bai-0023-2019

23. Sasakawa C. A new paradigm of bacteria-gut interplay brought through the study of Shigella. *Proc Japan Acad Ser B Phys Biol Sci*. 2010;86(3):229-243. doi:10.2183/pjab.86.229

24. Bonnans C, Chou J, Werb Z. Remodelling the extracellular matrix in development and disease. *Nat Rev Mol Cell Biol*. 2014;15(12):786-801. doi:10.1038/nrm3904

25. Neurath MF, Becker C, Barbulescu K. Role of NF-κB in immune and inflammatory responses in the gut. *Gut*. 1998;43(6):856-860. doi:10.1136/gut.43.6.856

26. Pasparakis M. Role of NF-κB in epithelial biology. *Immunol Rev*. 2012;246(1):346-358. doi:10.1111/j.1600-065X.2012.01109.x

27. Wu G, Fang YZ, Yang S, Lupton JR, Turner ND. Glutathione Metabolism and Its Implications for Health. *J Nutr*. 2004;134(3):489-492. doi:10.1093/jn/134.3.489

28. Imajo M, Ebisuya M, Nishida E. Dual role of YAP and TAZ in renewal of the intestinal epithelium. *Nat Cell Biol*. 2015;17(1):7-19. doi:10.1038/ncb3084

29. Pollard TD. The cytoskeleton, cellular motility and the reductionist agenda. *Nature*. 2003;422(6933):741-745. doi:10.1038/nature01598

30. Stempelj M, Kedinger M, Augenlicht L, Klampfer L. Essential Role of the JAK/STAT1 Signaling Pathway in the Expression of Inducible Nitric-oxide Synthase in Intestinal Epithelial Cells and Its Regulation by Butyrate. *J Biol Chem*. 2007;282(13):9797-9804. doi:10.1074/JBC.M609426200

31. Heneghan AF, Pierre JF, Kudsk KA. JAK-STAT and intestinal mucosal immunology. *JAK-STAT*. 2013;2(4). doi:10.4161/JKST.25530

32. Zapletal O, Tylichová Z, Neča J, et al. Butyrate alters expression of cytochrome P450 1A1 and metabolism of benzo[a]pyrene via its histone deacetylase activity in colon epithelial cell models. *Arch Toxicol*. 2017;3:2135-2150. doi:10.1007/s00204-016-1887-4

33. Goodman BE. Insights into digestion and absorption of major nutrients in humans. *Am J Physiol - Adv Physiol Educ*. 2010;34(2):44-53. doi:10.1152/advan.00094.2009

34. Gao J, Xu K, Liu H, et al. Impact of the gut microbiota on intestinal immunity mediated by tryptophan metabolism. *Front Cell Infect Microbiol*. 2018;8(FEB):13. doi:10.3389/fcimb.2018.00013

35. Keszthelyi D, Troost FJ, Masclee AAM. Understanding the role of tryptophan and serotonin metabolism in gastrointestinal function. *Neurogastroenterol Motil*. 2009;21(12):1239-1249. doi:10.1111/j.1365-2982.2009.01370.x

36. Alves A, Bassot A, Bulteau AL, Pirola L, Morio B. Glycine metabolism and its alterations in obesity and metabolic diseases. *Nutrients*. 2019;11(6):1356. doi:10.3390/nu11061356

37. Wang W, Wu Z, Dai Z, Yang Y, Wang J, Wu G. Glycine metabolism in animals and humans: Implications for nutrition and health. *Amino Acids*. 2013;45(3):463-477. doi:10.1007/s00726-013-1493-1

38. Konturek PC, Brzozowski T, Konturek SJ. Gut clock: Implication of circadian rhythms in the gastointestinal tract. *J Physiol Pharmacol*. Published online 2011.

39. Parkar SG, Kalsbeek A, Cheeseman JF. Potential role for the gut microbiota in modulating host circadian rhythms and metabolic health. *Microorganisms*. 2019;7(2):41. doi:10.3390/microorganisms7020041

40. Thelen K, Dressman JB. Cytochrome P450-mediated metabolism in the human gut wall. *J Pharm Pharmacol*. 2010;61(5):541-558. doi:10.1211/jpp.61.05.0002

41. Dallner G, Sindelar PJ. Regulation of ubiquinone metabolism. *Free Radic Biol Med*. 2000;29(3-4):285-294. doi:10.1016/S0891-5849(00)00307-5

42. Duszka K, Oresic M, May C Le, König J, Wahli W. PPARγ modulates long chain fatty acid processing in the intestinal epithelium. *Int J Mol Sci*. 2017;18(12):2559. doi:10.3390/ijms18122559

43. Wang J, Chao T, Wang G, et al. Transcriptome Analysis of Three Sheep Intestinal Regions reveals Key Pathways and Hub Regulatory Genes of Large Intestinal Lipid Metabolism. *Sci Rep*. 2017;7(1):1-12. doi:10.1038/s41598-017-05551-2

44. Kroetz DL, Zeldin DC. Cytochrome P450 pathways of arachidonic acid metabolism. *Curr Opin Lipidol*. Published online 2002. doi:10.1097/00041433-200206000-00007

45. Smith DM, Benaroudj N, Goldberg A. Proteasomes and their associated ATPases: A destructive combination. *J Struct Biol*. 2006;156(1):72-83. doi:10.1016/j.jsb.2006.04.012

46. Petrof EO, Claud EC, Sun J, et al. Bacteria-free solution derived from Lactobacillus plantarum inhibits multiple NF-kappaB pathways and inhibits proteasome function. *Inflamm Bowel Dis*. 2009;15(10):1537-1547. doi:10.1002/ibd.20930

47. Wahl MC, Will CL, Lührmann R. The Spliceosome: Design Principles of a Dynamic RNP Machine. *Cell*. 2009;136(4):701-718. doi:10.1016/j.cell.2009.02.009

48. Raveux A, Stedman A, Coqueran S, et al. Compensation between Wnt-driven tumorigenesis and cellular responses to ribosome biogenesis inhibition in the murine intestinal epithelium. *Cell Death Differ*. 2020;27(10):2872-2887. doi:10.1038/s41418-020-0548-6

49. Stedman A, Beck-Cormier S, Le Bouteiller M, et al. Ribosome biogenesis dysfunction leads to p53-mediated apoptosis and goblet cell differentiation of mouse intestinal stem/progenitor cells. *Cell Death Differ*. 2015;22(11):1865-1876. doi:10.1038/cdd.2015.57

50. Rodriguez MS, Dargemont C, Stutz F. Nuclear export of RNA. *Biol Cell*. 2004;96(8):639-655. doi:10.1016/j.biolcel.2004.04.014

51. Magallanes-Cruz PA, Flores-Silva PC, Bello-Perez LA. Starch Structure Influences Its Digestibility: A Review. *J Food Sci*. 2017;82(9):2016-2023. doi:10.1111/1750-3841.13809

52. Jang C, Hui S, Lu W, et al. The Small Intestine Converts Dietary Fructose into Glucose and Organic Acids. *Cell Metab*. 2018;27(2):351-361.e3. doi:10.1016/j.cmet.2017.12.016

53. Amrani N, Sachs MS, Jacobson A. Early nonsense: mRNA decay solves a translational problem. *Nat Rev Mol Cell Biol*. 2006;7(6):415-425. doi:10.1038/nrm1942

54. Clement SL, Lykke-Andersen J. No mercy for messages that mess with the ribosome. *Nat Struct Mol Biol*. 2006;13(4):299-301. doi:10.1038/nsmb0406-299

55. Brown G. Defects of thiamine transport and metabolism. *J Inherit Metab Dis*. 2014;37(4):577-585. doi:10.1007/S10545-014-9712-9/FIGURES/5

56. Lonsdale D. A Review of the Biochemistry, Metabolism and Clinical Benefits of Thiamin(e) and Its Derivatives. *Evidence-based Complement Altern Med*. 2006;3(1):49. doi:10.1093/ECAM/NEK009

57. Lee IH, Campbell CR, Cook DI, Dinudom A. Regulation of epithelial Na+ channels by aldosterone: Role of Sgk1. In: *Clinical and Experimental Pharmacology and Physiology*. Vol 35. John Wiley & Sons, Ltd; 2008:235-241. doi:10.1111/j.1440-1681.2007.04844.x

58. Fontana L, Partridge L, Longo VD. Extending healthy life span-from yeast to humans. *Science (80- )*. 2010;328(5976):321-326. doi:10.1126/science.1172539

59. Kenyon CJ. The genetics of ageing. *Nature*. 2010;464(7288):504-512. doi:10.1038/nature08980

60. Longo VD, Fontana L. Calorie restriction and cancer prevention: metabolic and molecular mechanisms. *Trends Pharmacol Sci*. 2010;31(2):89-98. doi:10.1016/j.tips.2009.11.004

61. Naidoo N. ER and aging-Protein folding and the ER stress response. *Ageing Res Rev*. 2009;8(3):150-159. doi:10.1016/j.arr.2009.03.001

62. Määttänen P, Gehring K, Bergeron JJM, Thomas DY. Protein quality control in the ER: The recognition of misfolded proteins. *Semin Cell Dev Biol*. 2010;21(5):500-511. doi:10.1016/j.semcdb.2010.03.006
